# Medical Care Situation of People with Myalgic Encephalomyelitis/Chronic Fatigue Syndrome in Germany

**DOI:** 10.3390/medicina57070646

**Published:** 2021-06-23

**Authors:** Laura Froehlich, Daniel B. R. Hattesohl, Leonard A. Jason, Carmen Scheibenbogen, Uta Behrends, Manuel Thoma

**Affiliations:** 1 Research Cluster D^2^L^2^, FernUniversität in Hagen, 58097 Hagen, Germany; 2 German Association for ME/CFS, 20146 Hamburg, Germany; daniel.hattesohl@dg.mecfs.de (D.B.R.H.); manuel.thoma@dg.mecfs.de (M.T.); 3 Center for Community Research, DePaul University, Chicago, IL 60614, USA; ljason@depaul.edu; 4 Institute of Medical Immunology, Charité University Medicine Berlin, 10117 Berlin, Germany; carmen.scheibenbogen@charite.de; 5 Department of Pediatrics, School of Medicine, Technical University of Munich, 80333 München, Germany; uta.behrends@mri.tum.de

**Keywords:** Myalgic Encephalomyelitis, Chronic Fatigue Syndrome, DePaul Symptom Questionnaire, medical care

## Abstract

*Background and Objective:* Myalgic Encephalomyelitis/Chronic Fatigue Syndrome (ME/CFS) is a severe illness with the hallmark symptom of Post-Exertional Malaise (PEM). Currently, no biomarkers or established diagnostic tests for ME/CFS exist. In Germany, it is estimated that over 300,000 people are affected by ME/CFS. Research from the United States and the UK shows that patients with ME/CFS are medically underserved, as they face barriers to medical care access and are dissatisfied with medical care. The first aim of the current research was to investigate whether patients with ME/CFS are medically underserved in Germany in terms of access to and satisfaction with medical care. Second, we aimed at providing a German-language version of the DePaul Symptom Questionnaire Short Form (DSQ-SF) as a tool for ME/CFS diagnostics and research in German-speaking countries. *Materials and Methods:* The current research conducted an online questionnaire study in Germany investigating the medical care situation of patients with ME/CFS. The questionnaire was completed by 499 participants who fulfilled the Canadian Consensus Criteria and reported PEM of 14 h or longer. *Results:* Participants frequently reported geographic and financial reasons for not using the available medical services. Furthermore, they reported low satisfaction with medical care by the physician they most frequently visited due to ME/CFS. The German version of the DSQ-SF showed good reliability, a one-factorial structure and construct validity, demonstrated by correlations with the SF-36 as a measure of functional status. *Conclusions:* Findings provide evidence that patients with ME/CFS in Germany are medically underserved. The German-language translation of the DSQ-SF provides a brief, reliable and valid instrument to assess ME/CFS symptoms to be used for research and clinical practice in German-speaking countries. Pathways to improve the medical care of patients with ME/CFS are discussed.

## 1. Introduction

The chronic illness Myalgic Encephalomyelitis or Chronic Fatigue Syndrome (we will use the acronym ME/CFS) is characterized by severe symptoms including profound exhaustion, muscle weakness and fatigability, pain, cognitive dysfunction, sleep disturbance, flu-like symptoms, and orthostatic intolerance [1,2,3]. The hallmark symptom of the illness is post-exertional malaise (PEM; i.e., the worsening of all symptoms after minimal exertion) [4,5]. To date, the etiology of ME/CFS is unknown, but the illness is associated with physiological abnormalities, e.g., an impaired energy metabolism [6,7], impaired cardiovascular function [8,9], as well as indicators of autoimmunity [10,11].

In the United States, it is estimated that 1.09 million adults (0.42% of the population) and 0.40 million children (0.75%) are affected by ME/CFS [12], and a meta-analysis of 46 studies conducted in 13 countries showed a pooled prevalence of 0.39% for adults [13]. A base rate of 0.4% would translate to 332,000 individuals affected by ME/CFS (including 54,000 children and adolescents) in Germany. The condition is largely unrecognized by health professionals and the public. In the United States, it is estimated that 84% of adults and 95% of children and adolescents with ME/CFS have not been diagnosed [14,15] and that ME/CFS results in annual costs between USD 35.9 and 50.9 billion in medical bills and lost incomes [12]. In the UK, an average yearly productivity loss due to employment discontinuation was estimated as GBP 22,684 per patient [16]. For the EU, an annual burden of EUROS 40 billion was estimated, although specific estimations of the cost of ME/CFS in Germany and further European countries are lacking [17]. ME/CFS is also an important health issue in children and adolescents [15], and severely affected patients in this age group often have difficulties completing their education due to ME/CFS symptoms [18].

### 1.1. Medical Care Situation of People with ME/CFS

Studies conducted in the United States have investigated the medical care situation of people with ME/CFS and showed that they are medically underserved [19,20] in that they lack equal access to healthcare [21]. First, people with ME/CFS report barriers to accessing medical care. These access barriers include geographical factors (e.g., low number of specialists in the area, not being able to travel large distances to see a specialist) as well as financial factors (e.g., cost for appointment not covered by insurance, travel to specialist too expensive) [20,22]. Second, people with ME/CFS report low satisfaction with the medical care they receive. The number of specialists who are knowledgeable about ME/CFS and regularly treat patients with this condition is low [23,24]. For example, Sunnquist, Nicholson, Jason, and Friedman [20] surveyed 898 US American individuals with self-reported ME/CFS; 52% of participants had never seen a specialist and only 11.5% were regularly treated by a specialist. Furthermore, 71% of participants saw four or more physicians in order to receive a diagnosis. Whereas participants who saw a specialist reported being satisfied with medical care, the satisfaction with care from non-specialists (e.g., GPs, staff of emergency departments) was reported to be low [20,24,25]. Timbol and Baraniuk [25] investigated the satisfaction with medical care in the emergency department (ED) in a sample of 282 patients with physician-diagnosed ME/CFS. Fifty-nine percent of patients reported having visited an ED in the past, predominantly due to orthostatic intolerance. Patients were dissatisfied with ED care in that they indicated that the staff were not knowledgeable about ME/CFS and half of the staff attributed patients’ complaints to stress, anxiety or psychological issues [25]. Other studies also showed that patients attributed their dissatisfaction with medical care to the inadequate training of physicians in treatment of patients with their illness [20,24].

Data on the access to and satisfaction with medical care of people with ME/CFS in Germany are currently lacking. If patients with ME/CFS in Germany faced similar barriers to medical care than in other countries and reported low satisfaction with medical care, this would indicate that they are also a medically underserved community. Based on research in the United States and the UK [16,20], patients with ME/CFS being medically underserved would be associated with individual and public financial losses also in Germany. Therefore, the first objective of the current research was to assess the medical care situation (i.e., access barriers and satisfaction with medical care) of people with ME/CFS in Germany.

### 1.2. Assessment of ME/CFS Symptoms

A second objective of the current research was to provide researchers and medical care personnel in German-speaking countries with a concise and time-efficient German-language questionnaire to assess and diagnose ME/CFS. Research points to multi-faceted causes of ME/CFS with 72% of patients reporting an infectious illness at the onset of the disease [26]. To date, there is no diagnostic biomarker or curative treatment [27,28,29]. However, during the last decades the DePaul Symptom Questionnaire (DSQ) has been developed as a valid and reliable psychometric instrument to assess ME/CFS symptoms [30]. The questionnaire has been translated into multiple languages and is available in several versions, including a time-efficient short form encompassing only 14 items (DePaul Symptom Questionnaire Short Form; DSQ-SF) [30,31]. It was designed to measure the frequency and severity of symptoms from all domains of the ME/CFS Canadian Consensus Criteria: Fatigue, PEM, sleep, pain, neurocognitive, autonomic, neuroendocrine, and immune symptoms [32]. The DSQ-SF has been shown to identify a relatively similar number of patients than the longer, 99-item DSQ-1 version, and reliably distinguishes between patients with ME/CFS, adult controls, and patients with multiple sclerosis [31]. Furthermore, a brief questionnaire to assess PEM, the hallmark symptom of ME/CFS, was recently developed [33]. The DePaul Symptom Questionnaire Post-Exertional Malaise (DSQ-PEM) can be used as an efficient and reliable screening instrument to identify PEM in patients with ME/CFS. The instrument showed high sensitivity and specificity in differentiating between patients with ME/CFS and other fatiguing illnesses, namely multiple sclerosis and post-polio syndrome [33].

Thus, the second aim of the current research was to provide a translation of the DSQ-SF and the DSQ-PEM into German language. In the absence of biomarkers and established diagnostic tests, German versions of the questionnaires would provide a valuable tool for time-constrained research protocols to assess ME/CFS symptoms and for clinical practice to diagnose patients with ME/CFS in Germany and other German-speaking countries. This would be an important step towards improving patients’ medical care situation.

## 2. Materials and Methods

### 2.1. Participants and Procedure

For the current project, we analyzed data collected for a superordinate research project [34], which was pre-registered at https://osf.io/spd9u/?view_only=bc79e0d225b9435caf6dd48fb6cd451b (accessed date: 22 June 2021). Participants with a self-reported diagnosis of ME/CFS were recruited via the four largest patient organizations for ME/CFS in Germany, their mailing lists, and social media. Data collection took place between May and June 2020. The online questionnaire took 30–45 min and was completed by 611 participants. We excluded participants who were under the age of 18 (*n* = 7) or did not consent to the inclusion of their data in the analyses (*n* = 3). Furthermore, we excluded participants who did not fulfil the Canadian Consensus Criteria for ME/CFS ([32]; *n* = 30; coded according to their responses to the DSQ-SF) [30]. Finally, as Cotler, Holtzman, Dudun, and Jason [33] showed that a duration of PEM longer than 14 h differentiated ME/CFS from other chronic diseases, we additionally excluded participants whose responses to the item “If you feel worse after activities, how long does this last” (item 9, DSQ-PEM) ranged between “1 h or less” and “11–13 h”; (*n* = 72). The final sample consisted of 499 participants.

After receiving information on data protection and the topic of the study, participants provided written consent in accordance with the EU General Data Protection Law, the research ethics guidelines of the American Psychological Association, as well as the Declaration of Helsinki. Then, they completed the DSQ-SF, DSQ-PEM, and SF-36, and provided information on demographics and illness history from the DSQ-2. Subsequently, they responded to items measuring their perceived barriers to medical care access and their satisfaction with medical care. For the superordinate research project, participants additionally completed measures of perceived causal attributions, perceived stigma, and satisfaction with social roles and activities (see pre-registration report, materials, and Froehlich, Hattesohl, Cotler, Jason, Scheibenbogen and Behrends [34] for a detailed description of these additional measures). Finally, participants were debriefed about the aims of the study and consented to the use of their data for analyses. The study received approval by the first author’s institutional ethics commission. Scales for which no official translations were available were translated from English to German by the project team and back-translated by a professional translator. Materials, data, and analysis scripts are available on the OSF [https://osf.io/5d8vu/ (use: 22 June 2021)]. The German translations of the DSQ-SF and the DSQ-PEM are displayed in Appendix A.

### 2.2. Materials

ME/CFS symptoms were assessed with the De Paul Symptom Questionnaire Short Form (DSQ-SF, 14 items) [31] and the DePaul Post-Exertional Malaise Questionnaire (DSQ-PEM; eight out of 10 items assessed; due to a programming error two items identical to the DSQ-SF were not assessed) [33]. Functional status was assessed with the Short-Form Health Survey (SF-36; 36 items) [35,36]. Furthermore, to assess access to medical care, participants were asked “Did you utilize any of these services in the past 6 months in regard to your ME/CFS? Primary care physician (GP), ME/CFS-specialist, neurologist, other specialist, hospital/stationary care, ME/CFS self-help, mental health, alternative medicine” and “Are there any services that you would like to use but are not accessible to you for one or more of the following reasons? Financial/insurance reasons, lack of knowledge of service availability (who treats my disease?), ME/CFS-associated impairment prevented access to service, travel distance and lack of transportation, no ME/CFS-specialist in geographic area, ME/CFS-specialist is not covered by health insurance, ME/CFS-specialist has a full waiting-list”, adapted from [20,22] to the characteristics of the German health-care system. Patient satisfaction with medical care was assessed with nine items (“Please indicate how satisfied you are with the care by your doctor that you are visiting most frequently because of ME/CFS”, e.g., “Overall, I feel satisfied with my appointments”, “Knowledgeable about symptoms/course of ME/CFS”, *1* = strongly disagree, *4* = strongly agree) [20]. In addition, participants indicated whether the doctor is a generalized or specialized physician (further indicating the area of specialty). Finally, participants completed items on demographics and illness history from the DSQ-2 (items 3–11; 94–99; 111–115, 116; [30]; demographics adapted to the German context).

### 2.3. Statistical Analyses

Statistical analyses were conducted with IBM SPSS version 26 and Mplus version 7 (confirmatory factor analysis only). The level of significance was α < 0.05, confidence intervals are displayed at the 95% level. Sample characteristics, health-related demographics and medical care access were investigated with descriptive statistics and frequency analysis. Multi-item measures (i.e., satisfaction with medical care, DSQ-SF) were aggregated to scales, as internal consistency was sufficient (Cronbach’s α > 0.80). Analyses of means was conducted with one-sample *t*-tests and paired-samples *t*-tests with bootstrapping (1000 samples). To investigate the factor structure of the DSQ-SF, we conducted the confirmatory factor analysis. Cutoffs for model fit statistics were CFI/TLI ≥ 0.90, RSMEA ≤ 0.08, and SRMR ≤ 0.05. Validity was investigated with correlational analyses, effect sizes were interpreted in accordance with Cohen (small effect: *r* = 0.10, moderate effect: *r* = 0.30, large effect: *r* = 0.50; [37]).

## 3. Results

### 3.1. Demographics

#### 3.1.1. Sample Characteristics

Of the total sample, 372 (74.5%) participants were female, 125 (25.1%) male, and two indicated “other” as their gender (0.4%). The age ranged between 18 and 76 years (*M* = 46.67, *SD* = 12.20). The majority of participants had German nationality (97%) and indicated Germany as their country of residence (99%). Participants had various levels of education, but a substantial part of the sample had higher (university) education (no degree: *n* = 5, 1.0%, Volks-/Hauptschulabschluss (primary school/secondary school): *n* = 22, 4.4%, Realschulabschluss/Mittlere Reife (secondary school leaving certificate): *n* = 118, 23.6%, Fachabitur/Fachhochschulreife (secondary school with qualification for technical university entrance): *n* = 60, 12.0%, Abitur/Allgemeine Hochschulreife (secondary school with qualification for university entrance): *n* = 80, 16.0%, university degree: *n* = 203, 40.7%, other: *n* = 10, 2.0%, one missing). Fifty-nine percent of participants reported being on disability, whereas 17% were working part-time, 12% were unemployed, 8% retired, 6% working full-time, 6% students, and 5% homemakers (multiple answers possible).

#### 3.1.2. Health-Related Demographics

Ninety percent of participants (*n* = 450) reported that they have been diagnosed with ME/CFS. All participants indicated that their problem with fatigue/energy lasted at least 6 months, with the majority of participants reporting a duration of 2 years or longer (“How long ago did your problem with fatigue/energy begin?”, 6–12 months (*n* = 8, 1.6%), 1–2 years (*n* = 16, 3.2%), longer than 2 years (*n* = 377, 75.6%), had a problem with fatigue/energy since childhood or adolescence (*n* = 98, 19.6%)). In line with Salit [26], three quarters of the sample (*n* = 378) reported that their fatigue/energy-related illness started after they experienced an infectious illness.

### 3.2. Access to and Satisfaction with Medical Care

Results on service utilization (“Did you utilize any of these services in the past 6 months in regard to ME/CFS?”, multiple answers possible) showed that participants predominantly visited their primary care physician, used ME/CFS self-help services, and alternative medicine. To a lesser extent, they visited specialized physicians, used mental health services or visited the hospital with regards to ME/CFS (Table 1).

Concerning barriers to service access (“Are there any services that you would like to use but are not accessible to you for one or more of the following reasons?”), all items except “ME/CFS specialist has a full waiting list” were affirmed by more than half of participants. The main factors participants perceived as barriers to service access were geographical reasons (i.e., lack of ME/CFS specialists in the area and lack of transportation), financial or insurance reasons, as well as lack of information about services (Table 2).

The nine items measuring patient satisfaction with medical care were averaged to a scale (α = 0.92). On average, participants indicated that they were rather not satisfied with medical care by the doctor they most frequently visited due to ME/CFS (*M* = 2.36, 95% CI [2.29; 2.43], *SE* = 0.04) which was significantly below the scale midpoint of 2.5 (*t*(469) = 4.08, *SE* = 0.03, *p* < 0.001). Half of the sample (*n* = 252, 50.5%) indicated that they visited their GP most frequently due to ME/CFS, whereas 32.9% (*n* = 164) visited a specialized physician, and 16.2% (*n* = 81) indicated that they were currently not in treatment due to ME/CFS. The physicians’ areas of specialty most frequently stated by patients were neurology/psychiatry, general medicine, internal medicine, hygiene and environmental medicine, as well as hematology and oncology (a detailed frequency table can be found on the OSF). Furthermore, 123 participants (24.6%) indicated that they were in treatment by a physician specialized in ME/CFS. These participants completed the satisfaction items again with regards to the specialist (α = 0.92). Results showed that satisfaction with medical care by a ME/CFS specialist was higher than the scale midpoint (*M* = 3.16, 95% CI [3.05; 3.26], *SE* = 0.06; *t*(122) = 11.70, *p* < 0.001). Furthermore, participants in this subsample reported higher satisfaction with medical care by a ME/CFS specialist compared to care by a physician not specialized in ME/CFS (*M* = 2.87, 95% CI [2.74; 3.00], *SE* = 0.07, *t*(121) = 4.64, *p* < 0.001).

### 3.3. German Version of the DePaul Symptom Questionnaire Short Form

For the DSQ-SF, we created composite scores per item by averaging frequency and severity ratings and then multiplying them by 25 to create a scale ranging from 0 to 100 for ease of interpretation [30]. Table 3 displays descriptive statistics of the DSQ-SF items (frequency and severity displayed separately in their original metric ranging from 0 to 4).

The confirmatory factor analysis on the composite scores (Cronbach’s α = 0.83) showed that the fit of a single-factor model was acceptable (*χ^2^*(73) = 222.70, *p* < 0.001; RMSEA = 0.06, CFI = 0.92, TLI = 0.90, SRMR = 0.05) when correlated error terms of the following items were allowed: “bloating” and “irritable bowel problems”, “problems remembering things” and “difficulty paying attention for a long period of time”, “cold limbs” and “feeling hot or cold for no reason”, as well as “unrefreshed sleep” and “muscle pain”. Detailed results of the CFA can be found on the Open Science Framework.

To investigate the construct validity of the German translation of the DSQ-SF, we computed bivariate correlations of the scale with the functional status (as measured by the SF-36). Higher frequency and severity of ME/CFS symptoms was significantly associated with lower functional status on all subscales. High correlations (*r* > 0.58) were found with the subscales of physical functioning and bodily pain, whereas correlations with social functioning, general health, vitality, and mental health were moderate (0.41 > *r* > 0.25). Small correlations were found with role physical and role emotional (*r* < 0.18; Table 4).

## 4. Discussion

Myalgic Encephalomyelitis/Chronic Fatigue Syndrome (ME/CFS) is a severe and chronic illness for which currently no cure or biomarker exists. ME/CFS is associated with losses of income and economic productivity [4,16,20,38]. Based on a prevalence of 0.4% [12,13], it is estimated that 332,000 people (including 54,000 children and adolescents) in Germany are affected by ME/CFS. Evidence from the United States indicates that people with ME/CFS are medically underserved [20,24,25]. The current research shows that this is likely also the case in Germany. An online questionnaire was distributed by the four largest German ME/CFS patient organizations. The final sample consisted of 499 participants with self-reported ME/CFS who fulfilled the Canadian Consensus Criteria [32] and indicated experiencing post-exertional malaise of 14 h or longer [33]. All participants included in the final sample also fulfilled diagnostic criteria by the Institute of Medicine [4].

### 4.1. Patients with ME/CFS in Germany Are Medically Underserved

Results point in the direction that people with ME/CFS in Germany are severely impaired in terms of health and social, as well as economic functioning. Despite high levels of education, only less than one quarter of the sample reported working part-time or full-time, whereas more than half of the participants were on disability. This pattern shows that similar to other countries, Germany also suffers financial and economic losses due to people living with ME/CFS not being able to contribute to the labor market [12,16,20,38]. Due to the chronic nature of the illness, this is unlikely to change, as more than 95% of the sample reported having had problems with fatigue/energy for 2 years or longer.

Results on access to and satisfaction with medical care present further evidence that patients with ME/CFS are medically underserved in Germany. Most patients reported being treated by their primary care physician and only one third reported having seen a physician specialized in ME/CFS in the last 6 months. This is consistent with evidence from the United States, where the number of ME/CFS specialists was reported to be low [20,23,24]. Moreover, the majority of participants indicated using self-help and alternative medicine, but only 40% or less reported being in treatment by a neurologist or other specialized physician. This pattern might indicate that patients use alternative services in search of treatment, as they might feel that primary care and specialized physicians are not able to provide them with satisfactory medical care. This is underlined by a recent literature review and expert survey on GP knowledge and understanding of ME/CFS. Results showed that in different European countries, between one third and half of GPs did not accept ME/CFS as a genuine clinical entity and even when they did, they lacked confidence in diagnosing or managing it [39,40]. Furthermore, in line with results from Sunnquist, Nicholson, Jason, and Friedman [20] as well as Thanawala and Taylor [22], patients with ME/CFS in Germany also predominantly reported both geographical/logistic as well as financial/insurance reasons for not being able to use medical services more frequently. This pattern reflects that the number of physicians specializing in ME/CFS in Germany is too low and as a consequence, patients are required to travel long distances to visit ME/CFS specialists, which might be prevented by or even exacerbate their ME/CFS symptoms. Insurance barriers include that the current official diagnostic guidelines on fatigue in Germany [41] recommend cognitive-behavioral therapy and graded exercise therapy as treatment. Other medical care might not be covered by health insurance. Furthermore, not being able to work and suffering associated income losses might also contribute to financial barriers to service utilization.

Findings are further corroborated by results on satisfaction with medical care. Overall, satisfaction with medical care by the physician patients visited most frequently due to ME/CFS (in most cases, the primary care physician) was reported to be low. Only one third of participants reported having seen a physician specialized in ME/CFS in the last 6 months. However, this subsample was significantly more satisfied with the medical care they received from the ME/CFS specialist compared to the non-specialist care. This is in line with studies showing that the medical personnel is not knowledgeable about ME/CFS and often attributes ME/CFS symptoms to psychological causes, which leads to patients being dissatisfied with the medical care they receive [20,24,25,39,40]. To provide patients with ME/CFS in Germany with improved medical care, we conclude that a more frequent and detailed education of medical students, physicians, and other medical personnel in Germany about ME/CFS symptoms, diagnostic criteria, and treatment approaches is necessary [39].

Research has shown a link between severe viral infection and ME/CFS [42] and 75% of the current sample reported that they developed ME/CFS after an infectious illness. In light of the current COVID-19 pandemic, it is to be expected that people recovering from SARS-CoV-2 are at risk of developing ME/CFS [43,44]. For example, a recent study from Germany showed that half of the participants with chronic COVID-19 syndrome fulfilled the Canadian Consensus Criteria for ME/CFS 6 months post infection with SARS-CoV-2 [45]. The expected increase in ME/CFS cases in Germany and around the world due to the COVID-19 pandemic creates an urgency to improve the medical care situation of patients with ME/CFS by providing better care and adequate diagnostic instruments.

### 4.2. The German-Language DSQ-SF: A Reliable and Valid Instrument for Research and Clinical Practice

In the absence of diagnostic tests or biomarkers for ME/CFS [27,28], the DSQ has been developed based on the Canadian Consensus Criteria [32] to assess ME/CFS symptoms. The instrument is available in several versions and has been translated into a variety of languages [30]. The DSQ has demonstrated excellent psychometric properties including high reliability and validity, as well as high sensitivity and specificity to classify patients with ME/CFS [30,31]. The current research provides a German-language translation of the brief DSQ-SF, which encompasses only 14 items and is thus well-suited for time-sensitive research protocols and clinical practice to diagnose ME/CFS [31]. The German translation of the DSQ-SF showed high reliability, the expected single-factor structure, as well as construct validity. Higher scores on the frequency and severity of ME/CFS symptoms correlated negatively with all subscales of the SF-36 [35,36], an established instrument to assess the functional status. This means that stronger ME/CFS symptoms assessed by the German version of the DSQ-SF were associated with the patients’ lower functional status. The pattern of interrelations of the DSQ-SF with the subscales of the SF-36 reflects the most common symptoms of ME/CFS. The strongest correlations were found with the subscales of physical functioning and bodily pain, reflecting the hallmark symptoms of post-exertional malaise, fatigability, as well as muscle weakness and pain. Moderate correlations were found with social functioning, general health, vitality and mental health, reflecting that patients with ME/CFS are severely impaired in terms of societal and social participation (see [34] for a detailed analysis on the relation of perceived stigma due to ME/CFS and lower functional status). As an indicator of discriminant validity, only small correlations of the DSQ-SF were found with the subscales of role physical and role emotional. This result reflects that due to the chronic nature of the illness, participants might have found ways to cope with the impairment they experience due to their symptoms and the associated difficulties for social relationships. Taken together, the current research provides a novel German translation of the DSQ-SF to be used for research and clinical practice in German-speaking countries.

### 4.3. Limitations and Future Directions

A first limitation is that the current research investigated the medical care situation of people who responded to an online questionnaire measuring only self-reported ME/CFS. This convenience sample might not be representative for the general population of patients with ME/CFS in Germany. However, we took several measures to ensure that our sample reflects the situation of patients with ME/CFS in Germany as accurately as possible. First, the questionnaire was distributed via the four largest German patient organizations, their mailing lists, and social media, increasing the likelihood of reaching patients with ME/CFS. Second, we excluded participants who did not fulfill the Canadian Consensus Criteria and did not report post-exertional malaise of at least 14 h after exertion [32,33]. Relatedly, the educational level of the sample was very high (40% reported having a university degree). It might be possible that highly educated patients with ME/CFS were particularly able or likely to participate in the online study due to higher familiarity with online questionnaires or better technical equipment/digital literacy. A combination of online recruitment and face-to-face recruitment in hospitals/doctor offices would be ideal to avoid systematic recruitment bias. However, as data were collected during the first wave of the COVID-19 pandemic (May/June 2020), such a combined recruitment approach was not possible. Future research could include a sample with a physician-confirmed diagnosis, collect data via paper-pencil questionnaires, and compare the situation of patients with ME/CFS to that of healthy controls and/or patients with other fatigue-related illnesses (e.g., multiple sclerosis).

A second limitation is that the questionnaire study was correlational and cross-sectional. Therefore, we could not investigate the medical care situation and relationships of ME/CFS symptoms with the functional status over time. Third, the current study did not include a comparison group of healthy controls or patients with other chronic illnesses to differentiate patients with ME/CFS from others. Thus, we could not investigate the Receiver Operating Characteristic analysis to set thresholds for subscores to assist with the diagnosis of ME/CFS in Germany. Future studies should include longitudinal study designs, ME/CFS screening questions in population-representative samples, studies with comparison groups, as well as cross-national surveys to shed a more encompassing light on the medical care situation of people with ME/CFS in Germany and other countries.

Finally, due to a programming error the DSQ-PEM was not fully assessed in the current research. The two items “next day soreness or fatigue after non-strenuous, everyday activities” and “minimum exercise makes you physically tired” are identical in the DSQ-SF and the DSQ-PEM, but were assessed only once in the current study. We provided the German translation for the DSQ-PEM in Appendix A, but could not investigate its psychometric properties. In our analyses, we only used one item to determine the cutoff value of >14 h of PEM duration as an inclusion criterion for our sample. Future studies should investigate the validity and reliability of the German version of the DSQ-PEM, as well as its interrelations with the DSQ-SF and functional status.

## 5. Conclusions

Results of the current research raise concerns about the medical care situation of people with ME/CFS in Germany, showing the need for adequate education of physicians about ME/CFS, as well as a more specialized treatment of patients with ME/CFS. Furthermore, there is a need for instruments to diagnose ME/CFS to be used in research and clinical practice. The current research provides a German version of the well-established DSQ-SF in order to provide an instrument to assess ME/CFS symptoms reliably and validly in German-speaking countries.

## Figures and Tables

**Table 1:** Frequencies of service utilization within the past 6 months with regards to Myalgic Encephalomyelitis/Chronic Fatigue Syndrome (ME/CFS).

Service	Frequency	Percentage
Primary care physician (GP)	344	68.9%
ME/CFS self-help (telephone hotlines for ME/CFS information, ME/CFS e-mails, ME/CFS literature, ME/CFS self-help groups)	330	66.1%
Alternative medicine (herbal medicine, self-awareness, biofeedback, acupuncture)	277	55.5%
Other specialist	200	40.1%
Neurologist	190	38.1%
Mental health (counseling, psychiatric hospitalization)	169	33.9%
Physicians specializing in the treatment of people with ME/CFS	168	33.7%
Hospital/stationary care	77	15.4%

**Table 2:** Frequencies of perceived barriers to medical care access.

Barrier	Frequency	Percentage
No ME/CFS specialist in the geographic area	394	79.0%
Financial/insurance reasons	356	71.3%
Lack of knowledge of service availability (who treats my disease?)	331	66.3%
ME/CFS specialist is not covered by health insurance	287	57.5%
Travel distance and lack of transportation	278	55.7%
ME/CFS-associated impairment prevented access to service	270	54.1%
ME/CFS specialist has a full waiting list	191	38.3%

ME/CFS: Myalgic Encephalomyelitis/Chronic Fatigue Syndrome.

**Table 3:** Descriptive statistics of German-language DePaul Symptom Questionnaire Short Form (DSQ-SF) items.

	Frequency			Severity		
Items	*M* [95% CI]	*SE*	*α*	*M* [95% CI]	*SE*	*α*
1. Fatigue/extreme tiredness	3.35 [3.28; 3.42]	0.04	0.897	3.10 [3.04; 3.17]	0.03	0.896
2. Next day soreness or fatigue after non-strenuous, everyday activities	3.22 [3.13; 3.30]	0.04	0.894	3.22 [3.14; 3.29]	0.04	0.894
3. Minimum exercise makes you physically tired	2.79 [2.68; 2.90]	0.05	0.894	2.97 [2.87; 3.06]	0.05	0.894
4. Feeling unrefreshed after you wake up in the morning	3.51 [3.45; 3.58]	0.03	0.897	3.08 [3.02; 3.14]	0.03	0.895
5. Pain or aching in your muscles	2.58 [2.48; 2.70]	0.06	0.896	2.42 [2.33; 2.51]	0.05	0.895
6. Bloating	1.90 [1.79; 2.01]	0.05	0.898	1.71 [1.62; 1.80]	0.04	0.896
7. Problems remembering things	2.00 [1.91; 2.09]	0.05	0.897	2.12 [2.03; 2.21]	0.05	0.896
8. Difficulty paying attention for a long period of time	2.90 [2.82; 2.98]	0.04	0.896	2.79 [2.72; 2.87]	0.04	0.895
9. Irritable bowel problems	2.08 [1.96; 2.19]	0.06	0.896	1.98 [1.88; 2.09]	0.05	0.894
10. Feeling unsteady on your feet, as if you might fall	1.86 [1.75; 1.97]	0.05	0.893	2.11 [2.01; 2.22]	0.06	0.893
11. Cold limbs (e.g., arms, legs, hands)	2.19 [2.08; 2.30]	0.06	0.898	1.74 [1.65; 1.83]	0.05	0.895
12. Feeling hot or cold for no reason	2.10 [2.00; 2.19]	0.05	0.894	2.02 [1.92; 2.11]	0.05	0.894
13. Flu-like symptoms	2.19 [2.10; 2.30]	0.05	0.896	2.46 [2.37; 2.55]	0.05	0.896
14. Some smells, foods, medications or chemicals make you feel sick	1.81 [1.70; 1.94]	0.06	0.894	1.80 [1.70; 1.91]	0.06	0.894

*Notes.* Results are displayed in the original metric before transformations. Frequency was assessed on a scale from 0 = none of the time to 4 = all of the time. Severity was assessed on a scale from 0 = symptom not present to 4 = very severe. Chronbach’s αs indicate internal consistencies of the scale when the item is removed (complete scale before transformations: α = 0.899).

**Table 4:** Bivariate correlations of the German-language DSQ-SF with the functional status.

Scales	(1)	(2)	(3)	(4)	(5)	(6)	(7)	(8)	(9)	(10)
(1) DSQ-SF	-									
(2) Physical functioning	−0.60 ***	-								
(3) Role physical	−0.18 ***	0.14 **	-							
(4) Bodily pain	−0.58 ***	0.34 ***	0.12 *	-						
(5) General health	−0.39 ***	0.23 ***	0.13 **	0.26 ***	-					
(6) Vitality	−0.32 ***	0.23 ***	0.03	0.19 ***	0.29 ***	-				
(7) Social functioning	−0.41 ***	0.40 ***	0.13 **	0.20 ***	0.25 ***	0.25 ***	-			
(8) Role emotional	−0.10 *	−0.02	0.06	0.20 ***	0.06	0.12 **	0.06	-		
(9) Mental health	−0.25 ***	0.08	0.04	0.22 ***	0.25 ***	0.29 ***	0.25 ***	0.53 ***	-	
(10) Gender	0.12 **	−0.15 ***	−0.02	−0.09	0.02	−0.04	0.00	−0.01	0.07 *	-
(11) Age	0.02	−0.01	−0.00	−0.08	0.13 **	−0.04	0.02	−0.14 **	−0.09	0.11 *

*Notes.* * *p* < 0.05, ** *p* < 0.01, *** *p* < 0.001. Gender was coded 0 = male, 1 = female. Displayed coefficients are Pearson correlations. Higher scores on the DSQ-SF represent more frequent/severe ME/CFS symptoms. Higher scores on the SF-36 subscales represent higher functioning.

## Data Availability

Materials, data, and analysis scripts are available on the Open Science Framework [https://osf.io/5d8vu/ (use: 22 June 2021)].

## Appendix A

**Table A1:** German translation and original English version of the DePaul Symptom Questionnaire Short Form.

Bitte Geben Sie für jedes der folgenden Symptome die Häufigkeit und Schwere an.	For each Symptom below, Please Circle One Number for Frequency and One Number for Severity.
*Häufigkeit:* Innerhalb der letzten 6 Monate, wie oft hatten Sie dieses Symptom? Bitte geben Sie für jedes der untenstehenden Symptome eine Zahl an von: 0 = nie, 1 = manchmal, 2 = ca. die Hälfte der Zeit, 3 = meistens, 4 = immer	*Frequency:* Throughout the past 6 months, how often have you had this symptom? For each symptom listed below, circle a number from: 0 = none of the time, 1 = a little of the time, 2 = about half the time, 3 = most of the time, 4 = all of the time
*Schwere:* Innerhalb der letzten 6 Monate, wie stark hat Sie dieses Symptom Sie beeinträchtigt? Bitte geben Sie für jedes der untenstehenden Symptome eine Zahl an von: 0 = Symptom nicht vorhanden, 1 = mild, 2 = moderat, 3 = schwer, 4 = sehr schwer	*Severity:* Throughout the past 6 months, how much has this symptom bothered you? For each symptom listed below, circle a number from: 0 = symptom not present, 1 = mild, 2 = moderate, 3 = severe, 4 = very severe
Symptom	Symptom
1. Fatigue/extreme Müdigkeit	1. Fatigue/extreme tiredness
2. Am nächsten Tag Schmerzen oder Fatigue nach nicht anstrengenden, alltäglichen Aktivitäten	2. Next day soreness or fatigue after non-strenuous, everyday activities
3. Minimale Bewegung verursacht körperliche Erschöpfung	3. Minimum exercise makes you physically tired
4. Sich nicht erholt fühlen, nachdem man morgens aufwacht	4. Feeling unrefreshed after you wake up in the morning
5. Schmerzen in den Muskeln	5. Pain or aching in your muscles
6. Blähungen	6. Bloating
7. Probleme, sich an Dinge zu erinnern	7. Problems remembering things
8. Schwierigkeiten, über einen längeren Zeitraum aufmerksam zu sein	8. Difficulty paying attention for a long period of time
9. Reizdarmprobleme	9. Irritable bowel problems
10. Sich unsicher auf den Beinen fühlen, als wenn man hinfallen könnte	10. Feeling unsteady on your feet, as if you might fall
11. kalte Gliedmaßen (z.B. Arme, Beine, Hände)	11. Cold limbs (e.g., arms, legs, hands)
12. Gefühl von Wärme oder Kälte ohne Grund	12. Feeling hot or cold for no reason
13. Grippeartige Symptome	13. Flu-like symptoms
14. Einige Gerüche, Medikamente oder Chemikalien verursachen Unwohlsein	14. Some smells, foods, medications or chemicals make you feel sick

**Table A2:** German translation and original English version of the DePaul Symptom Questionnaire Post-Exertional Malaise.

**Bitte geben Sie für jedes der folgenden Symptome die Häufigkeit und Schwere an.**	**For Each Symptom below, Please Circle One Number for Frequency and One Number for Severity.**
*Häufigkeit:* Innerhalb der letzten 6 Monate, wie oft hatten Sie dieses Symptom? Bitte geben Sie für jedes der untenstehenden Symptome eine Zahl an von: 0 = nie, 1 = manchmal, 2 = ca. die Hälfte der Zeit, 3 = meistens, 4 = immer	*Frequency:* Throughout the past 6 months, how often have you had this symptom? For each symptom listed below, circle a number from: 0 = none of the time, 1 = a little of the time, 2 = about half the time, 3 = most of the time, 4 = all of the time
*Schwere:* Innerhalb der letzten 6 Monate, wie stark hat Sie dieses Symptom Sie beeinträchtigt? Bitte geben Sie für jedes der untenstehenden Symptome eine Zahl an von: 0 = Symptom nicht vorhanden, 1 = mild, 2 = moderat, 3 = schwer, 4 = sehr schwer	*Severity:* Throughout the past 6 months, how much has this symptom bothered you? For each symptom listed below, circle a number from: 0 = symptom not present, 1 = mild, 2 = moderate, 3 = severe, 4 = very severe
1. Bleiernes Gefühl nach Bewegung	1. Dead, heavy feeling after starting to exercise
2. Am nächsten Tag Schmerzen oder Fatigue nach nicht anstrengenden, alltäglichen Aktivitäten	2. Next day soreness or fatigue after non-strenuous, everyday activities
3. Geistig müde nach der geringsten Anstrengung	3. Mentally tired after the slightest effort
4. Minimale Bewegung verursacht körperliche Erschöpfung	4. Minimum exercise makes you physically tired
5. Körperlich erschöpft oder krank nach leichter Aktivität	5. Physically drained or sick after mild activity
**Wählen Sie für jede der folgenden Fragen die Antwort, die Ihre PEM-Symptome am besten beschreibt.**	**For each question below, choose the answer which best describes your PEM symptoms.**
6. Wenn Sie nach der aktiven Teilnahme an außerschulischen Aktivitäten, Sport oder Ausflügen mit Freunden erschöpft wären, würden Sie sich innerhalb von ein oder zwei Stunden nach Beendigung der Aktivität erholen? 1 = Nein 2 = Ja	6. If you were to become exhausted after actively participating in extracurricular activities, sports or outings with friends, would you recover within an hour or two after the activity ended? 1 = No 2 = Yes
7. Erleben Sie eine Verschlechterung Ihrer Fatigue/auf Energie bezogenen Erkrankung nach minimaler körperlicher Anstrengung? 1 = Nein 2 = Ja	7. Do you experience a worsening of your fatigue/energy-related illness after engaging in minimal physical effort? 1 = No 2 = Yes
8. Erleben Sie eine Verschlechterung Ihrer Fatigue/auf Energie bezogenen Erkrankung nach geistiger Anstrengung? 1 = Nein 2 = Ja	8. Do you experience a worsening of your fatigue/energy-related illness after engaging in mental effort? 1 = No 2 = Yes
9. Wenn Sie sich nach Aktivität schlechter fühlen, wie lange dauert es an? 1 = ≤ 1 Stunde 2 = 2–3 Stunden 3 = 4–10 Stunden 4 = 11–13 Stunden 5 = 14–23 Stunden 6 = 1–2 Tage 7 = 3–7 Tage 8 = ≥ 7 Tage	9. If you feel worse after activities, how long does it last? 1 = ≤ 1 h 2 = 2–3 h 3 = 4–10 h 4 = 11–13 h 5 = 14–23 h 6 = 1–2 days 7 = 3–7 days 8 = ≥ 7 days
10. Wenn Sie sich nicht aktivieren, liegt es daran, dass Aktivität Ihre Symptome verschlimmert? 1 = Nein 2 = Ja	10. If you do not exercise, is it due to the fact that exercise makes your symptoms worse? 1 = No 2 = Yes
